# Vaping is associated with increased length of stay among cardiac inpatients

**DOI:** 10.18332/tid/207800

**Published:** 2025-07-30

**Authors:** Javad Heshmati, Kerri-Anne Mullen, Kathryn L. Walker, Hassan Mir

**Affiliations:** 1 Ottawa Model for Smoking Cessation, University of Ottawa Heart Institute, Ottawa, Canada; 2 School of Medicine, Faculty of Medicine, University of Ottawa, Ottawa, Canada; 3 School of Epidemiology and Public Health, University of Ottawa, Ottawa, Canada

**Keywords:** e-cigarette use, cardiovascular disease, hospital length of stay, vaping, healthcare utilization

## Abstract

**INTRODUCTION:**

The rapid increase in e-cigarette use, especially among youth, raises significant health concerns. Understanding their impact on high-risk populations, such as those with cardiovascular disease, is crucial for improving patient outcomes and reducing healthcare utilization. The aim of this study is to assess the impact of e-cigarette use on hospital length of stay (LOS) in patients with cardiovascular disease.

**METHODS:**

This cross-sectional survey was conducted at the University of Ottawa Heart Institute (November 2019–May 2020) among consecutive cardiology inpatients. Eligible participants were those admitted to the cardiac unit, fluent in French or English, and without cognitive or hearing impairments. The primary outcome is length of hospital stay. Data analysis included descriptive statistics and adjusted linear regression to explore e-cigarette use and hospital stay length, with significance set at p<0.05.

**RESULTS:**

Of 1616 cardiac patients, 1089 (73.0%) completed the survey. E-cigarette ever users were 10.4% (4.9% former, 5.5% current). Mean LOS was 11.03 days, longer for ever-users (13.1 days) than never-users (10.8 days). Ever users had a significantly longer LOS by 2.45 days (p=0.040), and current users by 3.24 days (p=0.039).

**CONCLUSIONS:**

E-cigarette use is associated with longer hospital stays among cardiac patients, even after adjusting for confounders. This underscores the potential harmful effects of vaping on cardiac recovery. Further research is needed to explore these associations and their implications for healthcare utilization.

## INTRODUCTION

There has been a rapid increase in e-cigarette use over the past decade^1^. In 2019, the prevalence of vaping was 24.5% in Canada and 25.4% in the USA^2,3^. There is growing evidence that e-cigarettes with nicotine can be used as a smoking cessation aid for those currently smoking cigarettes and interested in quitting by using e-cigarettes^4^. Further, recreational use, especially among youth and young adults, is a major concern given the high amounts of nicotine resulting in addiction and mental health disorders^5,6^. E-cigarette use has been associated with increased oxidative stress, endothelial dysfunction, and sympathetic activation, all of which are key mechanisms in the development of cardiovascular disease^7^. Population-based studies also suggest that individuals who use e-cigarettes may have a higher risk of myocardial infarction and other adverse cardiovascular outcomes compared to non-users^8^. Despite their known acute effects on the cardiorespiratory system, long-term outcomes are unclear^9^. As the popularity of e-cigarettes rises, it is increasingly important to understand their impact on health, especially among high-risk populations, such as those with cardiovascular disease^10^. Cardiovascular disease (CVD) is a leading cause of morbidity and mortality worldwide, imposing a significant burden on healthcare systems^11^. Patients with CVD often require prolonged hospitalization, which can be influenced by various factors including behavioral aspects, comorbidities, and adherence to treatment protocols^12^. Understanding the factors that contribute to extended hospital stays is crucial for improving patient outcomes and reducing healthcare utilization. Length of stay (LOS) in hospitals is not only a critical measure of healthcare efficiency but also a predictor of patient prognosis and overall recovery^13^. Although no studies, to date, have directly evaluated the impact of e-cigarette use on LOS among patients with cardiovascular disease, mechanisms well established in the literature such as endothelial dysfunction, oxidative stress, and vascular stiffening support a plausible link between e-cigarette exposure and prolonged cardiac hospitalizations. In this brief report, we aim to explore the association between vaping and LOS among cardiac inpatients at a tertiary-care hospital.

## METHODS

A cross-sectional survey was conducted at the University of Ottawa Heart Institute between November 2019 and May 2020, to examine characteristics among consecutive cardiology inpatients. The survey was administered in person during hospitalization or by telephone post-hospitalization. Eligibility criteria included admission to the cardiac inpatient unit during the study period, comprehension of French or English, lack of cognitive or hearing impairments, and sufficiently good health to participate. Survey questions were piloted for validity and reliability. The primary variable of interest for this secondary evaluation is self-reported use of e-cigarettes. Ever users are defined as current or former users of e-cigarettes. Those reporting use were asked about their patterns of use. Participants also responded to questions about demographic characteristics, past medical history, substance use, and their admitting diagnosis. Formal training was provided to study staff to follow standardized operating procedures in survey delivery. Participants were recruited through consecutive sampling based on admission date and time. Evaluation of cannabis use among this population was previously published^14^.

### Statistical analysis

Data analysis was performed using SPSS version 21.0. Descriptive statistics and variable distributions were examined for errors. Linear regression was used to explore relationships between e-cigarette use and length of stay in hospital. The adjustment was performed for variables including sex, age group, ethnicity, education level, employment status, admission diagnosis, ever tobacco use, physical activity and history of mental health diagnoses. Adjusted p-values and 95% confidence interventions (CIs) are reported, with statistical significance set at p<0.05. This project was approved as a Quality Initiative by the Ottawa Health Science Network Research Ethics Board (approval number QI-33) and consent was obtained from all survey participants.

## RESULTS

Of 1616 cardiac patients admitted, 1492 were eligible and 1089 (73.0%) completed the survey. Of these, 89.6% had never used e-cigarettes and 10.4% had ever used an e-cigarette (4.9% former and 5.5% current). The majority (56.6%) were aged ≥65 years, and 69.5% were male. Of the sample, 71.4% had used tobacco and 42.1% had used cannabis either currently or in past. Alcohol consumption varied, with 47.2% abstaining and 11% drinking more than 10 drinks per week.

The mean LOS was 11.03 days but varied based on e-cigarette use: 13.1 days for ever e-cigarette users (13.9 days for current and 12.1 days for former) and 10.8 days for those who had never used an e-cigarette. Complete data are presented in Table 1. Those who have used e-cigarettes have a significantly longer LOS by 2.45 days compared to never vapers (adjusted coefficient=2.45; 95% CI: 0.11–4.79, p=0.040). Similarly, current e-cigarette users have a significantly longer LOS by 3.24 days compared to former and never e-cigarette users (adjusted coefficient=3.24; 95% CI: 0.016–6.33, p=0.039). The association was adjusted by confounder variables as described in Table 1. Among these confounders, employment, diagnosis at admission, and alcohol intake significantly affect the correlation.

**Table 1:** Descriptive statistics and linear regression results for length of stay among cardiology inpatients by e-cigarette use status: Cross-sectional study, University of Ottawa Heart Institute, November 2019 to May 2020 (N=1089)

	*Total* *n (%)*	*Length of stay ( days )*	Correlation with length of stay*		
		*Mean (SD)*	*Coefficient*	*p*	*95 % CI*
**Total**	1089 (100)	11.03 (11.32)			
**E-cigarette use**					
Ever	112 (10.4)	13.05 (12.48)	**2.447^a^**	**0.040**	**0.106–4.789**
Never	969 (89.6)	10.82 (11.18)			
**Age** ( years )					
≤44	49 (4.5)	11.90 (14.66)	0.148	0.828	-1.187–1.484
45–64	424 (38.9)	10.80 (12.13)			
≥65	616 (56.6)	11.13 (10.41)			
**Sex**					
Male	757 (69.5)	10.45 (10.71)	0.495	0.538	-1.082–2.072
Female	332 (30.5)	12.36 (12.50)			
**Ethnicity**					
Other	118 (10.8)	10.08 (11.71)	1.045	0.378	-1.280–3.370
Caucasian	950 (87.2)	11.12 (11.26)			
**Education level**					
Bachelor’s or higher	336 (30.9)	10.38 (11.65)	0.113	0.569	-0.276–0.503
University lower than Bachelor’s	49 (4.5)	9.61 (11.58)			
College diploma	213 (19.6)	11.32 (11.07)			
Apprenticeship or trades certificate	52 (4.8)	10.38 (11.17)			
High school diploma	301 (27.6)	11.46 (10.73)			
No certificate, diploma or degree	121 (11.1)	12.18 (12.43)			
**Employment**					
Unemployed/on disability insurance	126 (11.6)	14.12 (15.55)	**1.679**	**0.005**	**0.521–2.837**
Employed	326 (29.9)	9.13 (8.82)			
Retired	617 (56.7)	11.25 (10.88)			
**Admission diagnosis**					
Coronary artery disease	665 (61.1)	9.84 (9.43)	**0.860**	**<0.001**	**0.462–1.258**
Peripheral artery disease	20 (1.8)	12.20 (12.55)			
Valvular disease	169 (15.5)	11.85 (11.54)			
Arrhythmia	59 (5.4)	8.10 (8.15)			
Heart failure	82 (7.5)	20.06 (20.34)			
Other	94 (8.6)	11.77 (9.94)			
**Ever diagnosed with a mental health condition**					
No	815 (74.8)	10.74 (10.47)	0.262	0.769	-1.485–2.008
Yes	242 (22.2)	12.07 (13.95)			
**Ever used any form of tobacco?**					
No	305 (28)	10.90 (12.70)	-0.376	0.656	-2.031–1.279
Yes	778 (71.4)	11.11 (10.76)			
**Have you ever used cannabis?**					
No	630 (57.9)	11.32 (12.05)	-0.441	0.579	-2.001–1.120
Yes	459 (42.1)	10.64 (10.22)			
**Alcohol** (drinks/week)					
None	514 (47.2)	12.78 (13.02)	**-0.746**	**0.012**	**-1.325 – -0.167**
≤5	313 (28.7)	9.92 (9.92)			
6–10	128 (11.8)	9.58 (9.27)			
11–15	62 (5.7)	7.42 (5.17)			
16–20	18 (1.7)	7.33 (6.77)			
>20	39 (3.6)	10.92 (10.94)			
**Substance use history**					
No	804 (73.8)	10.69 (10.91)	0.947	0.286	-0.795–2.689
Yes	219 (20.1)	12.14 (12.39)			
**Days of moderate or more intense exercise per week**					
None	290 (26.6)	12.45 (13.19)	-0.538	0.059	-1.097–0.021
1–2	52 (4.8)	11.00 (10.43)			
3–4	217 (19.9)	10.32 (11.07)			
>4	521 (47.8)	10.60 (10.38)			

* Results of linear regression adjusted for all presented variables.

a Linear regression performed for ever vaper (current and former) compared to never vaper.

## DISCUSSION

This evaluation is the first to report a significant relationship between e-cigarette use and increased length of stay in hospital among cardiac patients. Those who have never vaped had the shortest LOS followed by former e-cigarette users and then by current e-cigarette users. This graded effect suggests a dose–response relationship, which strengthens causal inference. This effect remains significant after adjustment for known confounders to hospitalization, underscoring the robustness of this association. These confounders themselves can influence cardiac health and recovery times, indicating a complex interplay between behavioral factors and vaping.

A possible mechanism for this is the known association of e-cigarette use with harmful cardiovascular and respiratory effects. Vaping has been linked to increased oxidative stress and inflammation. There are also known toxic substances in e-cigarette aerosols, such as nicotine, propylene glycol, and flavoring agents which have been shown to cause endothelial dysfunction, increased blood pressure, and heart rate^15^. Combined, these may exacerbate cardiac conditions and impede recovery, thereby increasing hospital stays. Animal models further demonstrate microvascular injury following chronic vapor exposure, providing biological plausibility for our clinical observations^16^. There may be other mechanisms that have not been elucidated at this time.

Cardiovascular disease is a leading cause of hospitalization in Canada and the US costing billions of dollars of direct and indirect costs per year. Prevention and cessation of e-cigarette use would be expected to improve clinical outcomes while reducing healthcare utilization. Of note, overall length of stay was higher in our sample than shown in previous studies, which may be due to the COVID-19 pandemic^17^.

### Limitations

It is important to consider these findings in the context of the limitations of this study, most notably the potential for selection bias due to the inclusion of only hospitalized cardiac patients, which may limit the generalizability of the findings to the broader population. Additional limitations include the small sample size, the cross-sectional observational design, and the potential misclassification of exposure due to concurrent use of e-cigarettes and combustible tobacco. Therefore, causal inferences cannot be made. Moreover, as the analysis adjusted for ‘ever tobacco use’ but not ‘current tobacco use’, residual confounding may persist, particularly given the high likelihood of co-use and the importance of modeling relevant confounders accurately. It is also possible that some patients switched to e-cigarettes due to more severe cardiovascular disease, suggesting reverse causality. However, these findings highlight the need to further evaluate the impact of e-cigarettes on LOS specifically within hospitalized cardiac patients, rather than extrapolating to cardiovascular outcomes in the general population. Future studies with longitudinal designs and more robust control for confounding are needed to better assess the association between vaping and clinical outcomes.

## CONCLUSIONS

E-cigarette use appears to be associated with increased length of stay in hospital among cardiac inpatients. This adds to the growing literature highlighting adverse effects of e-cigarette use. Although our findings are based on observational data, we recognize the importance of applying rigorous causal inference frameworks to enhance the validity of our conclusions. Future studies should consider approaches such as target trial emulation and other best practices outlined in recent methodological guidelines to better estimate causal effects of e-cigarette use on cardiovascular outcomes^18^.

## Data Availability

The data supporting this research are available from the authors on reasonable request.
